# Two New 3,4;9,10-*seco*-Cycloartane Type Triterpenoids from *Illicium difengpi* and Their Anti-Inflammatory Activities

**DOI:** 10.1155/2013/942541

**Published:** 2013-05-15

**Authors:** Chuntong Li, Fengmin Xi, Junling Mi, Zhijun Wu, Wansheng Chen

**Affiliations:** ^1^Department of Pharmacy, Shanghai Changzheng Hospital, Second Military Medical University, Fengyang Road 415, Shanghai 200003, China; ^2^School of Pharmacy, Chengdu University of Traditional Chinese Medicine, Liutai Road 1166, Chengdu, Sichuan 611137, China

## Abstract

A pair of new 3,4;9,10-*seco*-cycloartane type triterpenoid stereoisomerides: 24R,25-dihydroxy-3,4;9,10-*seco*-4(28)-cycloarten-10,3-olide (**1**) named Illiciumolide A and 24S,25-dihydroxy-3,4;9,10-*seco*-4(28)-cycloarten-10,3-olide (**2**) named Illiciumolide B were isolated from the stem bark of *Illicium difengpi*, as well as five known biogenetically related triterpenoids, including sootepin E (**3**), betulinic acid (**4**), lupeol (**5**), (all-*Z*)-1,5,9,13,17,21-hexamethyl-1,5,9,13,17,21-cyclotertracosahexaene (**6**), and (all-*E*)-2,6,10,15,19,23-hexamethyl-2,6,10,14,18,22-tetracosahexaene (**7**). The structures of two new compounds were determined on the basis of spectroscopic analysis including 1D-, 2D-NMR, and MS techniques. Two assays were conducted: inhibition of tumor necrosis factor-alpha (TNF-*α*) and inhibition of nuclear factor kappa B (NF-*κ*B) in RAW264. 7 cells induced by lipopolysaccharide (LPS). It was observed that compounds **1**, **2** and **7** showed significant inhibition of TNF-*α* production and NF-*κ*B release. The molecule docking results showed that compounds **1** and **2** got high fitness scores with dual specificity mitogen-activated protein kinase kinase 1 (MPKK1), whose activation plays a pivotal role between TNF-*α* and activation of NF-*κ*B. The anti-HIV-1 potency of compounds **1**–**5** was also discussed, in addition to the results of computer-aided screening for targets.

## 1. Introduction

Natural products have been, and continue to be, a major source of pharmacologically active substances from which drugs can be developed [1]. Medicinal plants that can relieve rheumatism, chills, and pains according to traditional Chinese medicine theory are fit for use by rheumatism sufferers. From these plants, many constituents with proved anti-inflammatory activity have been isolated and their structures were determined by spectroscopic methods.

*I. difengpi* (Illiciaceae), the stem bark of which has been applied for treatment of rheumatoid arthritis as a traditional Chinese medicine, is a small shrub growing in mountain areas of Guangxi province in China. *I. difengpi* is listed in Chinese Pharmacopoeia. In previous phytochemical investigation of barks of *I. difengpi* thirty compounds were isolated including dominant phenylpropanoids and neolignans [2–4] together with four sesquiterpene lactones [5] and three triterpenoids [6]. The anti-inflammatory activities of several neolignans were assayed by measuring the inhibitory ratio of *β*-glucuronidase release in rat PMNs induced by PAF *in vitro *[2, 5]. The sesquiterpene lactones were predicted to exhibit neurotrophic activity [7–9]. However, there have been limited studies that focus on the triterpenoids from *I. difengpi* and of the family Illiciaceae. Until now, only six cycloartanes [6, 10, 11] were reported to be isolated from family Illiciaceae, while from family Schisandraceae (*Schisandra* and *Kadsura, *order Illiciales) more than 150 triterpenoids [12] have been isolated. In particular, they both contain *seco*-cycloartane triterpenoids. These characteristic chemical structures from the Schisandraceae and their activities were widely studied. Most of the *seco-*cycloartanes from the family Schisandraceae were demonstrated to possess anti-HIV-1 activity. 

As a part of our study to find the active constituents, an investigation of *I. difengpi* was undertaken, leading to isolation and structural elucidation of two new 3,4;9,10-*seco*-cycloartane triterpenoids and three known triterpenoids together with two squalenes. In order to shed some light on biological activities, the anti-inflammatory and anti-HIV-1 properties of the isolates were evaluated and discussed.

## 2. Materials and Methods

### 2.1. General Experimental Procedures

Optical rotations were measured using a Perkin-Elmer 341 polarimeter. IR spectra were recorded on a NEXUS 470 FT-IR spectrometer (Thermo Nicolet, USA). 1D (^1^H, ^13^C, DEPT) and 2D (COSY, NOESY, HSQC, HMBC) NMR spectra were acquired on a Bruker Avance 600 NMR spectrometer operating at 600 (^1^H) or 150 (^13^C) MHz using the residual solvent signals as an internal reference (CDCl_3_ *δ*_H_ 7.26 ppm, *δ*_C_ 77.0 ppm). NMR samples were in 3 mm Shigemi tubes during NMR analyses. High-resolution mass spectrometric data were obtained on an Agilent 6220 TOF LC/MS instrument (Agilent Technologies, MA, USA) with ESI ionization in the positive mode. Column chromatography (CC) was performed on Sephadex LH-20 gel (40–70 *μ*m, Amersham Pharmacia Biotech AB, Uppsala, Sweden), *YMC*-GRL ODS-A (50 *μ*m; *YMC*, MA, USA), and silica gel H (100–200 and 200–300 mesh, Qingdao Haiyang Chemical Co. Ltd., Qingdao, China). TLC analyses were performed on Si_60_F_254_ plates and visualized under UV light or by heating after spraying with 10% H_2_SO_4_/EtOH solution. Semipreparative RP-HPLC isolation was achieved with an Agilent 1200 instrument using a *YMC *5 *μ*m C18 column (250 mm × 10 nm) eluted with 80% MeCN/H_2_O at 1-2 mL/min. Peak detection was made with a refractive index detector (RID). The positive controls in the anti-inflammatory assay were tripterygium tablets (TRT) and total glucosides of paeony (TGP). Purity was assessed by HPLC and determined to be 95% or greater for compounds **1**–**7** at the time of testing.

### 2.2. Plant Material

The stem barks of *Illicium difengpi* were purchased from Caitongde Pharmacy, Shanghai, China, in January 2010. Plant material was authenticated by Professor Lianna Sun (Department of Pharmacognosy, School of Pharmacy, Second Military Medical University) based on morphological characters. Voucher specimen (No. 20100110) has been deposited at the Herbarium of Department of Pharmacognosy, School of Pharmacy, Second Military Medical University, Shanghai, China.

### 2.3. Extraction and Isolation

The air-dried stem bark of* I. difengpi* (40 kg) was powdered and extracted three times with 80% ethanol under reflux. The solvent was concentrated to obtain a crude extract (1200 g) which was suspended in water (10 L) and extracted with petroleum ether (10 L × 3), EtOAc (10 L × 3), and BuOH (10 L × 3), affording 40, 560, and 300 g of each dried fraction, respectively. 

The dried petroleum ether fraction (Fr._1_) (40 g) was chromatographed on silica gel column (CC) (80 × 5 cm, gradient with petroleum ether: EtOAc = 100 : 0→0 : 100) to give eight main fractions (Fr._1-1_–Fr._1−8_), among which Fr._1-2_ gave compound **7** (27 mg), Fr._1−7_ provided compound **6** (85 mg), and Fr._1−8_ afforded compound **3** (34 mg) through Sephadex LH-20 CC (150 × 2 cm, CH_2_Cl_2_: MeOH = 1 : 1). The EtOAc extract (Fr._2_) (560 g) was chromatographed on silica gel CC (150 × 10 cm, gradient with CH_2_Cl_2_: MeOH = 300 : 1→0 : 100) to give four main fractions (Fr._2-1_–Fr._2−4_). Fr._2-2_ was subjected to silica gel CC (20 × 2 cm, gradient with petroleum ether: EtOAc = 100 : 1→1 : 1) affording a mixture of compounds **4** and **5**. The mixture was rechromatographed using silica ODS-A gel CC (20 × 2 cm, gradient with MeOH: H_2_O = 1 : 1→1 : 0) to give pure compounds **4** (45 mg) and **5** (21 mg). Fr._2-3_ was fractionated by silica gel CC (35 × 3 cm, gradient with petroleum ether: EtOAc = 100 : 1→5 : 1) to yield three subfractions (Fr._2-3-1_–Fr._2-3-3_). Fr._2-3-3_ was rechromatographed using semipreparative HPLC system (Agilent 1200 series; *YMC* HPLC C18 column-5 *μ*m, 250 × 10 mm, refractive index detector; flow 2mL/min; mobile phase MeCN: H_2_O = 80 : 20) to give pure compounds **1** (8 mg) and **2** (2.5 mg).

### 2.4. Characterization of Compounds

Compound **1**: yellowish solid; [*α*]_*D*_^25^ + 41.2 (*c *0.5, MeOH); IR (KBr) *ν*_max⁡_ 3402, 2929, 2872, 1763, 1458, 1377, 1273, 1238, 1194, 1172, 1074, 1041, 1007, 899 cm^−1^; ^1^H NMR and ^13^C NMR data see Table 1; HRESIMS *m/z* 475.3774 [M+H]^+^ (calcd. for C_30_H_51_O_4_, 475.3784).

 Compound **2**: yellowish solid; [*α*]_*D*_^25^ + 94.0 (*c *0.5, MeOH); IR (KBr) *ν*_max⁡_ 3454, 2931, 2873, 1764, 1461, 1452, 1379, 1259, 1195, 1171, 1080, 1039, 1009, 920, 870 cm^−1^; ^1^H NMR and ^13^C NMR data see Table 1; HRESIMS *m*/*z* 475.3765 [M+H]^+^ (calcd. for C_30_H_51_O_4_, 475.3784).

### 2.5. Inhibition of TNF-*α* Release Assay

Isolated compounds were tested for their ability to inhibit TNF-*α* release from LPS-stimulated RAW 264.7 macrophages using enzyme-linked immunosorbent assay (ELISA) as a quantitative assay. Tripterygium tablets (TRT) and total glucosides of paenia (TGP) were used as positive controls. The ELISA Max set standard (BioLegend, San Diego, CA, USA) was performed according to the manufacturer's instruction strictly. The inhibition ratio (IR) was calculated as IR (%) = (*A*_LPS_ − *A*_S_)/*A*_LPS_ × 100%, where *A*_LPS_ and *A*_S_ refer to the amount of TNF-*α* in cells pretreated with LPS and samples, respectively.

### 2.6. Inhibition of NF-*κ*B Release Assay

The effect of compounds **1–7** on NF-*κ*B production from LPS-treated RAW264.7 cells was monitored. Tripterygium tablets (TRT) and total glucosides of paeony (TGP) were used as positive controls. Cells were placed on 96-well plates (Costar) at a density of 1.0 × 10^5^ cells mL^−1^, maintained in DMEM containing 10% FBS for 24 h. After changing the medium, the samples were incubated at 37°C with 5% CO_2_ for 4 h, and 1 *μ*g/mL LPS was then added and incubated for another 48 h. After 48 h, cells were collected and completely cracked using 1× lysis buffer. After centrifugation at 1500 rpm for 5 min, the supernatant was obtained. 100 *μ*L luciferase assay reagent and 20 *μ*L cell lysate were added to it in order rapidly. The detection results were read in 10 s. The IR was calculated in a similar way to the above formula, IR (%) = (*I*_LPS_ − *I*_S_)/*I*_LPS_ × 100%, where *I*_LPS_ and *I*_S_ refer to the fluorescence intensity of cells pretreated with LPS and samples, respectively.

### 2.7. Statistical Analysis

The statistical significance of differences was determined by two-tailed Student's *t*-test for unpaired data.

## 3. Results and Discussion

### 3.1. Structure Analysis

**Figure 1:**
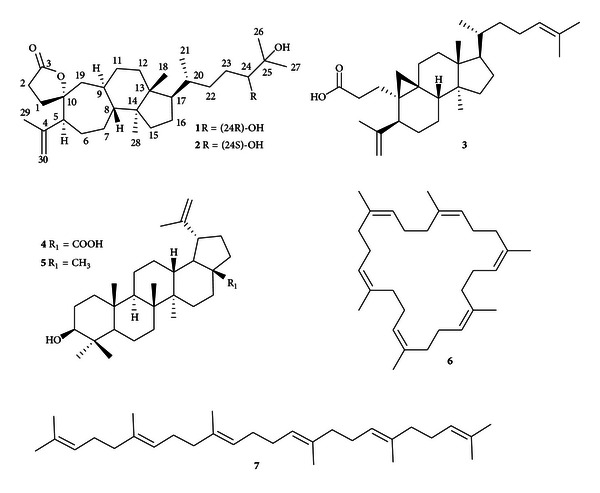
Structures of compounds **1**–**7**.

**Figure 2:**
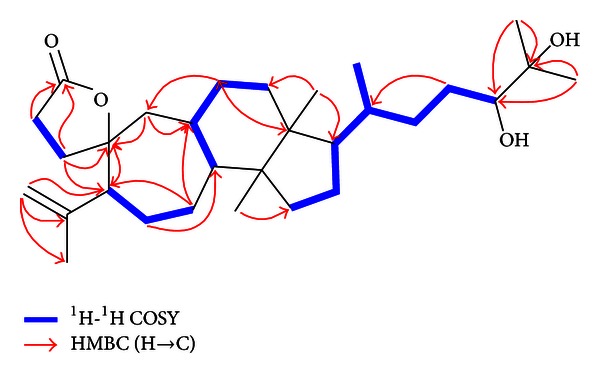
Key ^1^H-^1^H COSY (bold lines) and HMBC (H→C) correlations of compound **1**.

**Figure 3:**
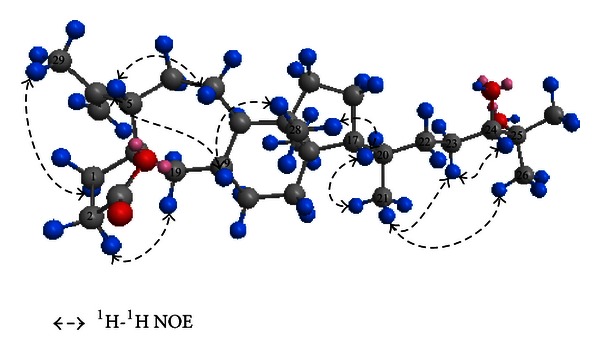
Key NOE correlations of compound **1**.

Illiciumolide A (**1**) was isolated as a yellowish solid. The positive HRESI-MS analysis showed a pseudo-molecular ion at *m*/*z* 475.3774 [M+H]^+^, consistent with the formula C_30_H_50_O_4_ (calcd. 474.3709), which accounted for six degrees of unsaturation. IR (KBr) absorption bands were observed as 3402 cm^−1^ (OH), 2929 cm^−1^ (CH_3_), 1763 cm^−1^ (C=O), 1377 cm^−1^ (CH_2_), and 899 cm^−1^ (C=CH_2_). The ^13^C NMR and DEPT spectra exhibited thirty resonances, including six quaternary carbons, six methines, twelve methylenes, and six methyls. Among these, five primary methyls (*δ*_C_ 14.7, H_3_-18; *δ*_C_ 16.6, H_3_-28; *δ*_C_ 22.6, H_3_-29; *δ*_C_ 23.2, H_3_-26; *δ*_C_ 26.5, H_3_-27) and a secondary methyl (*δ*_C_ 18.3, H_3_-21) were determined as evident from their multiplicities in the ^1^H NMR spectrum (Table 1). The ^13^C NMR spectrum of **1** revealed the presence of one lactone carbonyl carbon (*δ*_C_ 177.3) and a pair of olefinic carbons (*δ*_C_ 146.6, *δ*_C_ 115.0). Taking into consideration the presence of a pair of methylene protons (*δ*_H_ 4.80 and 4.91) in the ^1^H NMR spectrum of **1** (Table 1), these features indicated the existence of a terminal methylene group in accordance with the IR spectrum absorption. The oxygenated quaternary carbon appeared at the low field *δ*_C_ 91.7, together with the lactone carbonyl carbon (*δ*_C_ 177.3), suggesting that the C-3 (*δ*_C_ 177.3) may lactonize to C-10 (*δ*_C_ 91.7) forming a five-membered lactone ring, which required future analysis of HMBC and ^1^H-^1^H COSY. Apart from two degrees of unsaturation occupied by one double bond and one carbonyl, the remaining four degrees of unsaturation indicated that **1** should possess a tetracyclic system. Detailed comparison of the ^1^H and ^13^C NMR spectra of **1** with those of schisanterpene A [13] suggested a similar structure for rings A–D in both compounds and might be derived from cycloartane type triterpenoids, but a pair of double bonds between C-24 and C-25 and the carboxyl group at C-26 in schisanterpene A was absent in **1**, which instead exhibited vicinal diol (C-24, *δ*_C_ 78.7; C-25, *δ*_C_ 73.2) and two methyls (C-26, *δ*_C_ 23.2; C-27, *δ*_C_ 26.5). As a result, **1** was tetracyclic and belonged to 3,4;9,10-*seco*-type triterpenoids [13, 14]. Subsequently, the structure was fully elucidated by 2D NMR spectroscopy. The H_2_-1, H_2_-2, and H-5 showed distant correlations with C-3, C-10, and C-19, coupled with ^1^H-^1^H COSY correlation of H-1/H-2 (Figure 2), consistent with the lactone ring (A) substructure. A detailed analysis of its HSQC, HMBC and ^1^H-^1^H COSY spectra confirmed that **1** contained a seven-membered ring (B) evident as the HMBC correlations from H-5 to C-10, from H-6 to C-8, from H-7 to C-5, and C-9 and from H-19 to C-9 and C-10, as well as ^1^H-^1^H COSY correlations of H-5/H-6/H-7 and H8/H-9 (Figure 2). The structure of rings C and D was deduced from the ^1^H-^1^H COSY correlations of H-9/H-11/H-12 and H-15/H-16/H-17 and HMBC correlations from H_3_-13 to C-8 and C-15, H_3_-14 to C-12 and C-17, and H-11 to C-19 and C-14. From the above deduction, compound **1** and schisanterpene A were confirmed to have the similar structure in rings A–D, while the chain from C-17 was verifiably different. Further evidence supporting chain structure was provided by the presence of the correlations of H-15/H-16, H-21/H-20, H-22/H-23, and H-23/H-24 as deduced from the COSY spectrum and HMBC correlation from H-23 to C-20. Furthermore, the vicinal diol with two methyls termination was proved from the HMBC evident correlations from H_3_-26 (H_3_-27) to C-24 and C-25 (Figure 2). The relative stereochemistry of compound **1** was deduced from NOESY correlations (Figure 3). The oxygen atom of the spiroring on C-10 was in the *α*-orientation, as the NOESY correlations between H-2*β* and H_2_-19 and H-1*β* and H_3_-29 are the same as those of schinalactone B. Correlations between H-5*α* with H-7*α* and H-9 clearly showed that these protons were on the same face. Other important NOESY correlations were observed between H-9 and H-11*α* and H_3_-28, and H-7*α*/H-16*α*, indicating that H_3_-28 was *α*-orientation while H_3_-18 was on the other side. Finally about the C-17 side chain, the intense cross-peaks between H-16*α*, H-17, and H_3_-21 suggested that both H-17 and C-21 have *α* orientation in **1**. Except for 24-OH, the relative configurations of **1** were the same as schinalactone B. The 24-OH configuration was deduced by the resonances of protons and carbons at C-23, C-24, and C-25 and by the *J* values between H-23 and H-24 (*J* = 6.2 Hz). In the previous literature, *J* value between H-23 and H-24 in 24R-configuration was reported to be around 6.5 and 1.0 Hz, while that in 24S-configuration was around 10.5 and 1.9 Hz [15, 16]. Comparison with the literature data and significant NOE correlations between H-24 and H-23*α*, alone with modeling in Chem3D 11.0 (Cambridge Soft, Inc.) as shown in Figure 3, both suggested 24-OH was R-configuration. Based on these evidences, the structure of **1** was determined to be 24R,25-dihydroxy-3,4;9,10-*seco*-4(28)-cycloarten-10,3-olide (Figure 1).

Illiciumolide B (**2**) was obtained as a yellowish solid with molecular formula C_30_H_50_O_4_ established by positive HRESI-MS (*m*/*z* 475.3765 [M+H]^+^, calcd. for 475.3784). Both compounds **1** and **2** have the same molecular formula, suggesting that they are isomer. The ^1^H and ^13^C NMR spectra of **1** were quite similar to those of **2**. In comparison with their ^1^H and ^13^C NMR data (Table 1), it was found that the chemical shifts of C-20, C-21, C-22, C-23, and C-24 were slightly different, while the other chemical shifts remained unchanged. These suggested that compound **2** was a stereoisomer of **1**, which was confirmed by the NOE spectrum. In the NOE experiments, correlation signal from H-24 to H-23*β* (*δ*_H_ 1.09–1.14) in **2** was observed, while H-24 to H-23*α* (*δ*_H_ 1.36) in compound **1** was observed. Besides, the *J* value between H-23 and H-24 (*J* = 9.5, 1.6 Hz) was different with that (*J* = 6.2 Hz) of compound **1**. By comparison with the literature [15, 16], compound **2** was deduced to be 24S-configuration. Therefore, compound **2** was elucidated as 24S,25-dihydroxy-3,4;9,10-*seco*-4(28)-cycloarten-10,3-olide (Figure 1). The spectra of Illiciumolide A and B are presented in Supplementary Materials available online at http://dx.doi.org/10.1155/2013/942541, including MS, IR, and NMR spectra.

In addition to the two new compounds, the known triterpenoids **3** [15, 17, 18], **4** [19, 20], and **5** [21, 22], and squalenes **6** [23] and **7** [24] were also isolated from the *I. difengpi*. This is the first report on isolation of compounds **3**, **6,** and **7** and 3,4-*seco-* and 3,4;9,10-*seco-*type triterpenoids from *I. difengpi*.

### 3.2. Bioactivities Analysis

#### 3.2.1. Inhibition of TNF-*α* Release Assay

**Figure 4:**
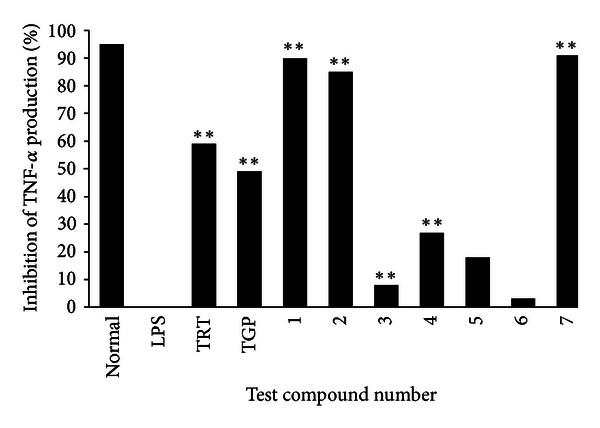
Inhibitory rate of TNF-*α* production from LPS-stimulated RAW 264.7 cells by compounds **1**–**7** at a concentration of 25 mg/mL. **P < 0.01 for TNF-*α* levels in RAW 264.7 cells treated with LPS in the presence of the test compounds versus that in the absence of the test compounds.

The anti-inflammatory activities of compounds **1**–**7** at 25 *μ*g/mL were assessed by determining the inhibitory ratio of TNF-*α* release in LPS-stimulated RAW 264.7 macrophages *in vitro*. Tripterygium tablets (TRT) and total glucosides of paenia (TGP) were used as positive controls. As shown in Figure 4, the concentrations of TNF-*α* in the RAW 264.7 cells pretreated with compounds **1**, **2,** and **7** were reduced by 90%, 85%, and 91%, respectively, compared to LPS-stimulated RAW 264.7 cells, while the inhibitory rates of two positive controls TRT and TGP were 59% and 49%, respectively. These results demonstrated that compounds **1**, **2,** and **7** had a significant inhibitory effect on TNF-*α* release from macrophages.

#### 3.2.2. Inhibition of NF-*κ*B Release Assay

**Figure 5:**
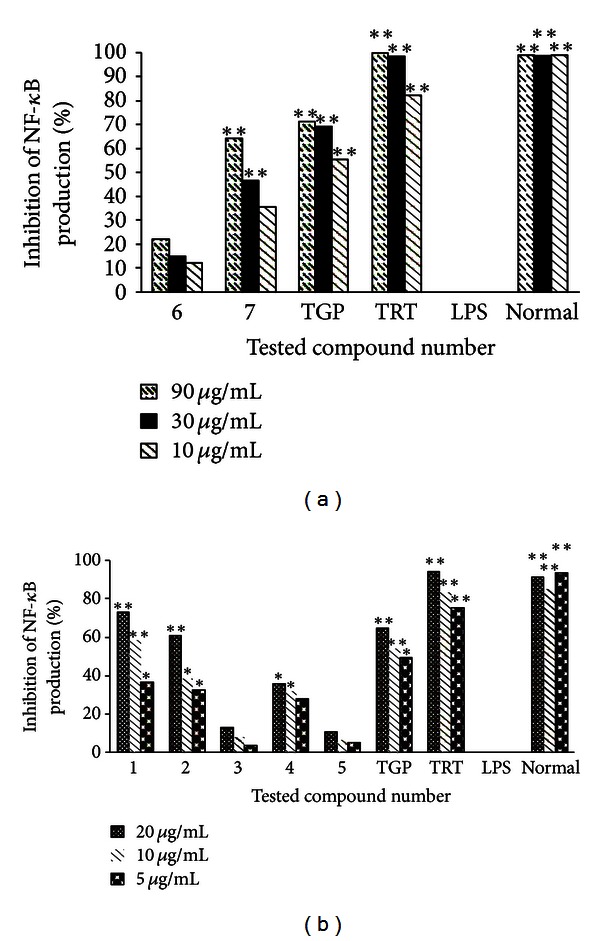
Inhibitory rate of NF-*κ*B production from LPS-stimulated RAW 264.7 cells by (a) Compounds **6**-**7** at three concentrations 90 *μ*g/mL, 30 *μ*g/mL, and 10 *μ*g/mL. (b) Compounds **1**–**5** at three concentrations 20 *μ*g/mL, 10 *μ*g/mL, and 5 *μ*g/mL. *P < 0.05 and **P < 0.01 for TNF-*α* levels in RAW 264.7 cells treated with LPS in the presence of the test compounds versus that in the absence of the test compounds.

Based on the results obtained from TNF-*α* release experiments, compounds **1**–**7** were further assessed for their possible effect on NF-*κ*B production from RAW 264.7 cells stimulated with LPS. The cytotoxic effects of tested compounds on LPS-stimulated RAW 264.7 cells were determined initially. The results showed that compounds **1**–**5** did not affect cell viability at concentrations up to 25 *μ*g/mL and **6** and **7** did up to 100 *μ*g/mL. Compound **1** at concentration 10 *μ*g/mL and 20 *μ*g/mL, **2** at 20 *μ*g/mL, and **7** at 90 *μ*g/mL greatly reduced the NF-*κ*B production stimulated by LPS (*P* < 0.01). It was observed that these compounds showed a dose-dependent inhibition of NF-*κ*B release in LPS-stimulated RAW264.7 cells. The remaining compounds showed slight activities against NF-*κ*B release (Figure 5).

#### 3.2.3. Molecule Docking Screening for Targets

Binding properties for compounds **1**, **3**, **4**, and **5** on various inflammation related ligands were estimated by computer-aided molecular docking. The results showed that all these compounds had good binding with dual specificity mitogen-activated protein kinase kinase 1 (MPKK1), whose activation is involved in the upstream of NF-*κ*B signal pathway [1], followed by production of many proinflammatory cytokines as well as other important inflammation-released proteins (see Table 2).

Similar computer-aided molecule docking to screen targets had been calculated and several ligands, including gag-pol polyprotein, protease, androgen receptor, and renin, exhibited high fit score and norm fit score (Table 3).

### 3.3. Discussion

This is the first time that 3,4;9,10-*seco*-type cycloartane triterpenoids were isolated from *I. difengpi *and from genus *Illicium*. There has been only five cycloartane triterpenoids [10], two of which were 3,4-*seco*-cycloartane type triterpenoids [11], isolated from family Illiciaceae. These *seco*-cycloartane types of triterpenoids have been extensively isolated from family Schisandraceae, especially 46 compounds of 3,4-*seco*-cycloartane type out of 166 triterpenoids totally [22]. It was widely accepted that family Illiciaceae has close relationship with theSchisandraceae (*Schisandra* and *Kadsura*) and both of them were under the Illiciales. The common possession of the *seco*-cycloartanes in both* I. difengpi* and the Schisandraceae is of great significance.

*I. difengpi* is included in Chinese Pharmacopoeia for its traditional treatment of rheumatoid arthritis (RA). In RA patients, TNF-*α* levels are elevated in RA synovial fluid, serum, and synovial fibroblasts [25]. Macrophages are important cells implicated in the initiation of inflammatory responses, so the agents that inhibit TNF-*α* production have been studied almost exclusively in these cells. The objective of this present study was to investigate the potential activities against RA of triterpenoids isolated from the *I. difengpi* against RA. The main focus was to explore the attenuation of LPS-induced acute inflammatory response under *in vitro* conditions. Our research results suggested that triterpenoids **1**, **2**, and **7** may modulate macrophages responsiveness to LPS. These three compounds reduced the production of TNF-*α* stimulated with LPS. However, the molecular mechanisms of the induction of TNF-*α* production in RAW264.7 cells in response to LPS remain incompletely understood. Another important anti-inflammatory activity assay results showed that compounds **1**, **2**, and **7** have the potential effect on suppression of NF-*κ*B in a concentration-dependent manner in LPS-stimulated RAW 264.7 cells. These similar inhibitions on TNF-*α* and NF-*κ*B suggested some close relationship through some signal pathway. Previous studies have elucidated some signal pathways leading to TNF-*α* in response to LPS. In particular, the activation of NF-*κ*B may play a significant role in LPS-induced expression of TNF-*α* [26, 27]. We deduced that compounds **1**, **2**, and **7** may inhibit LPS-induced TNF-*α* production through inhibition of NF-*κ*B signal pathway; similar conclusion has been reported in triterpenoids anti-inflammatory activity studies [28]. It was reported that the activation of MPKK1 is in the upstream of NF-*κ*B signal pathway [1]. The computer-aided molecule modeling results also showed compound **1** binding well with MPKK1. From this molecule docking, we further deduced that compounds **1** and **2** may interact with MPKK1 and consequently suppress the NF-*κ*B.

Some of the *seco*-cycloartanes have reported to have anti-HIV-1 activity [29] and anti-HBV (hepatitis B virus) activity [30]. The earliest example was nigranoic acid from stems of *Schisandra sphaerandra* that has been demonstrated to be capable of inhibiting HIV viral reverse transcriptase with IC_50_ = 74.1 *μ*g/mL [31]. Further structure-activation relation experiment were reported quite recently that cycloartane triterpenoids with *seco* structure in ring A shown to inhibit HIV integrase (IN), while cycloartane triterpenoids without *seco*-structure in ring A showed weak or no inhibition at all [32]. The *seco*-structure seems to be the crucial anti-HIV-1 activation related structural feature. Besides, betulinic acid and its derivatives were also extensively reported to have a potent inhibitory activity against human immunodeficiency virus type 1 (HIV-1) [33–36]. These reports suggest that compounds **1**–**5** may also possess certain antiviral activity. Molecule docking provided some well-binding targets, including gag-pol polyprotein, protease, androgen receptor and renin. Among these, gag-pol polyprotein and protease have close relation with HIV infection. These results may offer more clues for further experiments for anti-HIV-1 potential and their mechanism.

## 4. Conclusions

We reported the first *seco*-triterpenoids isolated from the stem barks of* I. difengpi*. Two new 3,4;9,10-*seco*-cycloartane triterpenoids, as well as three known triterpenoids and two biosynthetic related squalenes were structurally elucidated through spectral methods, together with comparison with literature. Our study has demonstrated the anti-inflammatory activity of these compounds. They showed remarkable anti-inflammatory activity, especially compounds **1**, **2**, and **7** and deserve further considerations towards developing as an effective anti-inflammatory drug. As reported in the previous literatures, some *seco*-cycloartane triterpenoids are capable of inhibiting HIV-1. The computer-aided molecule docking provided clues for targets screening and further mechanism research. Our study suggested that the triterpenoids from *I. difengpi* are of great interest as potential leads for natural product-based candidates for further studies.

## Supplementary Material

Figure S1: High resolution ESI mass spectrum of Illiciumolide A (1).
Figure S2: IR spectrum of Illiciumolide A (1).
Figure S3: ^1^H NMR spectrum of Illiciumolide A (1) in CD_3_Cl.
Figure S4: ^13^C NMR spectrum of Illiciumolide A (1) in CD_3_Cl.
Figure S5: ^1^H-^1^H COSY spectrum of Illiciumolide A (1) CD_3_Cl.
Figure S6: HSQC spectrum of Illiciumolide A (1) in CD_3_Cl.
Figure S7: HMBC spectrum of Illiciumolide A (1) in CD_3_Cl (H→C).
Figure S8: NOESY spectrum of Illiciumolide A (1) in CD_3_Cl.
Figure S9: High resolution ESI mass spectrum of Illiciumolide B (2).
Figure S10: IR spectrum of Illiciumolide B (2).
Figure S11: ^1^H NMR spectrum of Illiciumolide B (2) in CD_3_Cl.
Figure S12: ^13^C NMR spectrum of Illiciumolide B (2) in CD_3_Cl.
Figure S13: ^1^H-^1^H COSY spectrum of Illiciumolide B (2) CD_3_Cl.
Figure S14: HSQC spectrum of Illiciumolide B (2) in CD_3_Cl.
Figure S15: HMBC spectrum of Illiciumolide B (2) in CD_3_Cl (H→C).
Figure S16: NOESY spectrum of Illiciumolide B (2) in CD_3_Cl.

## Figures and Tables

**Table 1:** ^
1^H-NMR (600 MHz) and ^13^C-NMR (150 MHz) data for compounds **1** and **2** (CDCl_3_, *δ*_H_ in ppm, *J* in Hz).

Position	**1**		**2**	
	*δ*_C_, mult.	*δ*_H_	*δ*_C_, mult.	*δ*_H_
1	31.5, CH_2_	2.33 (m, H_b_-1) 1.77–1.84 (m, H_a_-1)	31.5, CH_2_	2.30–2.36 (m, H_b_-1) 1.70–1.76 (m, H_a_-1)
2	29.6, CH_2_	2.41–2.48 (m, H_a_-2) 2.41–2.48 (m, H_b_-2)	29.6, CH_2_	2.42–2.48 (m, H_a_-2) 2.42–2.48 (m, H_b_-2)
3	177.3, qC	—	177.3, qC	—
4	146.6, qC	—	146.6, qC	—
5	54.8, CH	2.54 (d, 9.6, H-5)	54.8, CH	2.56 (d, 9.8, H-5)
6	31.4, CH_2_	1.65–1.70 (m, H_a_-6) 1.82–1.89 (m, H_b_-6)	31.4, CH_2_	1.61–1.67 (m, H_a_-6) 1.82–1.89 (m, H_b_-6)
7	30.5, CH_2_	1.46–1.51 (m, H_a_-7) 1.79–1.83 (m, H_b_-7)	30.5, CH_2_	1.41–1.46 (m, H_a_-7) 1.72–1.77 (m, H_b_-7)
8	48.4, CH	1.43 (dd, 3.2, 7.0, H-8)	48.4, CH	1.42–1.47 (m, H-8)
9	31.7, CH	1.90 (td, 7.4, 14.0, H-9)	31.7, CH	1.85–1.92 (m, H-9)
10	91.7, qC	—	91.7, qC	—
11	29.6, CH_2_	1.23 (m, H_a_-11) 1.73 (m, H_b_-11)	29.6, CH_2_	1.46 (m, H_a_-11) 1.73 (m, H_b_-11)
12	32.7, CH_2_	1.61–1.64 (m, H_a_-12) 1.75–1.80 (m, H_b_-12)	32.7, CH_2_	1.61–1.64 (m, H_a_-12) 1.75–1.80 (m, H_b_-12)
13	45.5, qC	—	45.5, qC	—
14	49.2, qC	—	49.2, qC	—
15	33.3, CH_2_	1.06–1.14 (m, H_a_-15) 1.23–1.28 (m, H_b_-15)	34.0, CH_2_	0.97–1.02 (m, H_a_-15) 1.23–1.25 (m, H_b_-15)
16	27.9, CH_2_	1.37–1.42 (m, H_a_-16) 1.92–1.98 (m, H_b_-16)	27.8, CH_2_	1.47 (m, H_a_-16) 1.92–1.98 (m, H_b_-16)
17	51.0, CH	1.49–1.53 (m, H-17)	50.9, CH	1.49–1.55 (m, H-17)
18	14.7, CH_3_	0.80 (s, H_3_-18)	14.7, CH_3_	0.80 (s, H_3_-18)
19	49.2, CH_2_	1.64–1.70 (m, H_a_-19) 1.77–1.83 (m, H_b_-19)	49.2, CH_2_	1.64–1.70 (m, H_a_-19) 1.78–1.83 (m, H_b_-19)
20	35.9, CH	1.37–1.47 (m, H-20)	36.4, CH	1.51–1.55 (m, H-20)
21	18.3, CH_3_	0.86 (d, 7.22, H_3_-21)	18.6, CH_3_	0.88 (d, 6.5, H_3_-21)
22	33.1, CH_2_	1.13–1.20 (m, H_a_-22) 1.35–1.40 (m, H_b_-22)	33.3, CH_2_	1.13–1.20 (m, H_a_-22) 1.35–1.40 (m, H_b_-22)
23	28.3, CH_2_	1.36 (m, H_a_-23) 1.14–1.22 (m, H_b_-23)	28.6, CH_2_	1.36 (m, H_a_-23) 1.09–1.14 (m, H_b_-23)
24	78.7, CH	3.31 (t, 6.2, H-24)	79.6, CH	3.27 (dd, 9.5, 1.6, H-24)
25	73.2, qC	—	73.2, qC	—
26	23.2, CH_3_	1.14 (s, H_3_-26)	23.2, CH_3_	1.15 (s, H_3_-26)
27	26.6, CH_3_	1.19 (s, H_3_-27)	26.5, CH_3_	1.21 (s, H_3_-27)
28	16.6, CH_3_	0.83 (s, H_3_-28)	16.7, CH_3_	0.84 (s, H_3_-28)
29	22.6, CH_3_	1.76 (s, H_3_-29)	22.6, CH_3_	1.77 (s, H_3_-29)
30	115.0, CH_2_	4.80 (s, H_a_-30) 4.91 (s, H_b_-30)	115.0, CH_2_	4.81 (s, H_a_-30) 4.92 (s, H_b_-30)

**Table 2:** Screening anti-inflammation targets over molecule docking of compounds **1–5**.

Compound number	Molecules docking results		
	Target name	Fit score	Norm fit score
	Dual specificity mitogen-activated protein kinase kinase 1 (MPKK1)	4.111	0.4567
	Glucocorticoid receptor (GR)	4.010	0.5012
	Prothrombin	3.843	0.4271
**1**/**2**	Alpha-1-antitrypsin	3.834	0.6390
	Glycogen synthase kinase-3 beta	3.786	0.5409
	ADAM 17	3.726	0.9315
	Tyrosine-protein kinase SYK	3.712	0.6187
	Protein kinase C theta type	3.701	0.5287

	Dual specificity mitogen-activated protein kinase kinase 1 (MPKK1)	5.171	0.5745
	Proto-oncogene tyrosine-protein kinase LCK	4.370	0.4370
**3**	Glucocorticoid receptor	4.332	0.5415
	Glycogen synthase kinase-3 beta	4.298	0.6140
	Cathepsin B	4.110	0.4566
	Peroxisome proliferator-activated receptor alpha	4.042	0.5053
	Prothrombin	3.988	0.6646

	Dual specificity mitogen-activated protein kinase kinase 1 (MPKK1)	3.660	0.4067
**4**	Proto-oncogene tyrosine-protein kinase LCK	3.596	0.5137
	Prothrombin	3.496	0.3885
	Leukotriene A-4 hydrolase	3.354	0.3727

	Dual specificity mitogen-activated protein kinase kinase 1 (MPKK1)	4.246	0.4718
	Glucocorticoid receptor	3.931	0.4913
	Estrogen receptor	3.728	0.5325
	Leukocyte elastase	3.709	0.4121
**5**	Protein kinase C theta type	3.689	0.5270
	Glycogen synthase kinase-3 beta	3.642	0.5203
	Proto-oncogene tyrosine-protein kinase LCK	3.626	0.6044
	Mitogen-activated protein kinase 10	3.596	0.5137
	Peroxisome proliferator-activated receptor alpha	3.576	0.4470

**Table 3:** Screening anti-HIV targets over molecule docking of compounds **1**–**5**.

Compound number	Molecules docking results		
	Target name	Fit score	Norm fit score
	Androgen receptor	4.294	0.7157
**1/2**	Gag-pol polyprotein	4.281	0.3058
	Glucocorticoid receptor	4.010	0.5012
	Renin	3.781	0.5401

	Androgen receptor	4.768	0.7947
**3**	Glucocorticoid receptor	4.332	0.5415
	Renin	4.121	0.5151

	Gag-pol polyprotein	3.549	0.2218
**4**	Androgen receptor	3.359	0.4199
	Renin	3.347	0.4183
	Thymidine kinase	3.337	0.4171

	Androgen receptor	4.022	0.5028
**5**	Glucocorticoid receptor	3.931	0.4913
	Gag-pol polyprotein	3.674	0.2625
	Protease	3.556	0.4446
