# Supplementary material for: Two New 3,4;9,10-seco-Cycloartane Type Triterpenoids from Illicium difengpi and Their Anti-Inflammatory Activities
Source: Evid Based Complement Alternat Med. 2013 May 15;2013:942541. doi: 10.1155/2013/942541 (PMC3671308; doi:10.1155/2013/942541)

Supporting Information

Two New 3,4;9,10-*seco* Cycloartane Type Triterpenoids from *Illicium difengpi* and their Anti-inflammatory Activities

Chuntong Li [1], Fengmin Xi [1], Junling Mi [2], Zhijun Wu [1]* and Wansheng Chen [1,] *

[1] Department of Pharmacy, Shanghai Changzheng Hospital, Second Military Medical University, Fengyang Road 415, Shanghai 200003, PR China;
E-Mails: chuntongli@yahoo.cn (C.L.); fengmin.x@gmail.com (F.X.); wuzhijun999@sina.com (Z.W.); chenwanshengsmmu@yahoo.com.cn (W.C.).

[2] School of Pharmacy, Chengdu University of Traditional Chinese Medicine, Liutai Road 1166, Chengdu, 611137, Sichuan, PR China; E-Mail:miboai@126.com (J.M.).

***** Author to whom correspondence should be addressed;
E-Mail: wuzhijun999@sina.com (Z.W.);
chenwanshengsmmu@yahoo.com.cn (W.C.)
Tel.: +86-021-81886181; Fax: +86-021-81886191.

Contents

S1. **Figure S1**. High resolution ESI mass spectrum of Illiciumolide A (**1**).

S2. **Figure S2**. IR spectrum of Illiciumolide A (**1**).

S3. **Figure S3**. ^{1}H NMR spectrum of Illiciumolide A (**1**) in $CDCl_3$.

S4. **Figure S4**. ^{13}C NMR spectrum of Illiciumolide A (**1**) in $CDCl_3$.

S5. **Figure S5**. ^{1}H-^{1}H COSY spectrum of Illiciumolide A (**1**) $CDCl_3$.

S6. **Figure S6**. HSQC spectrum of Illiciumolide A (**1**) in $CDCl_3$.

S7. **Figure S7**. HMBC spectrum of Illiciumolide A (**1**) in $CDCl_3$ (H→C).

S8. **Figure S8**. NOESY spectrum of Illiciumolide A (**1**) in $CDCl_3$.

S9. **Figure S9**. High resolution ESI mass spectrum of Illiciumolide B (**2**).

S10. **Figure S10**. IR spectrum of Illiciumolide B (**2**).

S11. **Figure S11** ^{1}H NMR spectrum of Illiciumolide B (**2**) in $CDCl_3$.

S12. **Figure S12**. ^{13}C NMR spectrum of Illiciumolide B (**2**) in $CDCl_3$.

S13. **Figure S13**. ^{1}H-^{1}H COSY spectrum of Illiciumolide B (**2**) $CDCl_3$.

S14. **Figure S14**. HSQC spectrum of Illiciumolide B (**2**) in $CDCl_3$.

S15. **Figure S15**. HMBC spectrum of Illiciumolide B (**2**) in $CDCl_3$ (H→C).

S16. **Figure S16**. NOESY spectrum of Illiciumolide B (**2**) in $CDCl_3$.

Figure S1. High resolution ESI mass spectrum of Illiciumolide A (**1**).

Qualitative Analysis Report

**Data Filename**	DF-62-POS-1.d	**Sample Name**	DF-62
**Sample Type**	Sample	**Position**	P1-F3
**Instrument Name**	Instrument 1	**User Name**	
**Acq Method**	TEST-POS-03.m	**Acquired Time**	10/16/2012 3:35:36 PM
**IRM Calibration Status**	Success	**DA Method**	ERROR.m
**Comment**			

User Spectra

Fragmentor Voltage	Collision Energy	Ionization Mode
80	0	Esi

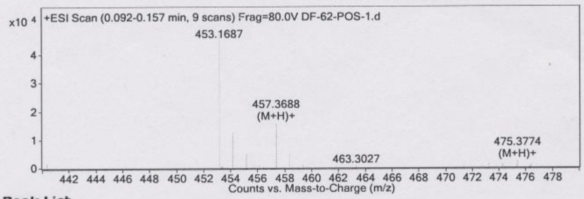

Peak List

m/z	z	Abund	Formula	Ion
256.2642	1	11680		
274.2749	1	20861		
284.2951	1	14099		
288.2911	1	26833		
415.2133	1	39301		
437.1946	1	39742		
453.1687	1	47103		
454.1715	1	12177		
457.3688	1	15863	C30 H49 O3	(M+H)+
922.0097	1	12109		

Formula Calculator Element Limits

Element	Min	Max
C	0	100
H	0	367
O	0	25
N	0	0

Formula Calculator Results

Formula	Best	Mass	Tgt Mass	Diff (ppm)	Ion Species	Score
C30 H48 O3	TRUE	456.3615	456.3603	-2.59	C30 H49 O3	95.86
C30 H50 O4	TRUE	474.37	474.3709	1.98	C30 H51 O4	69.43

--- End Of Report ---

Figure S2. IR spectrum of Illiciumolide A (**1**).

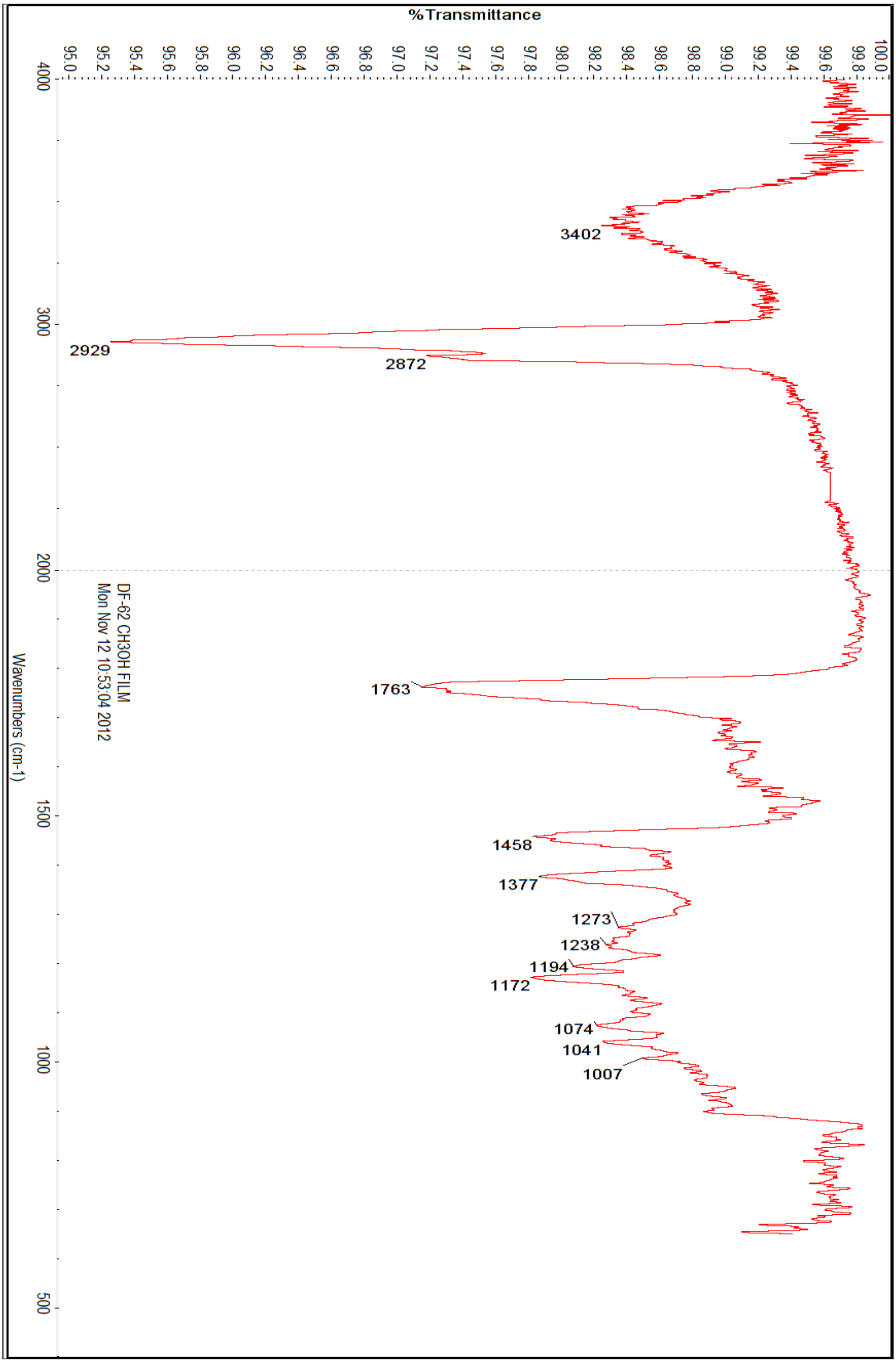

Figure S3. ^{1}H NMR spectrum of Illiciumolide A (**1**) in $CDCl_3$.

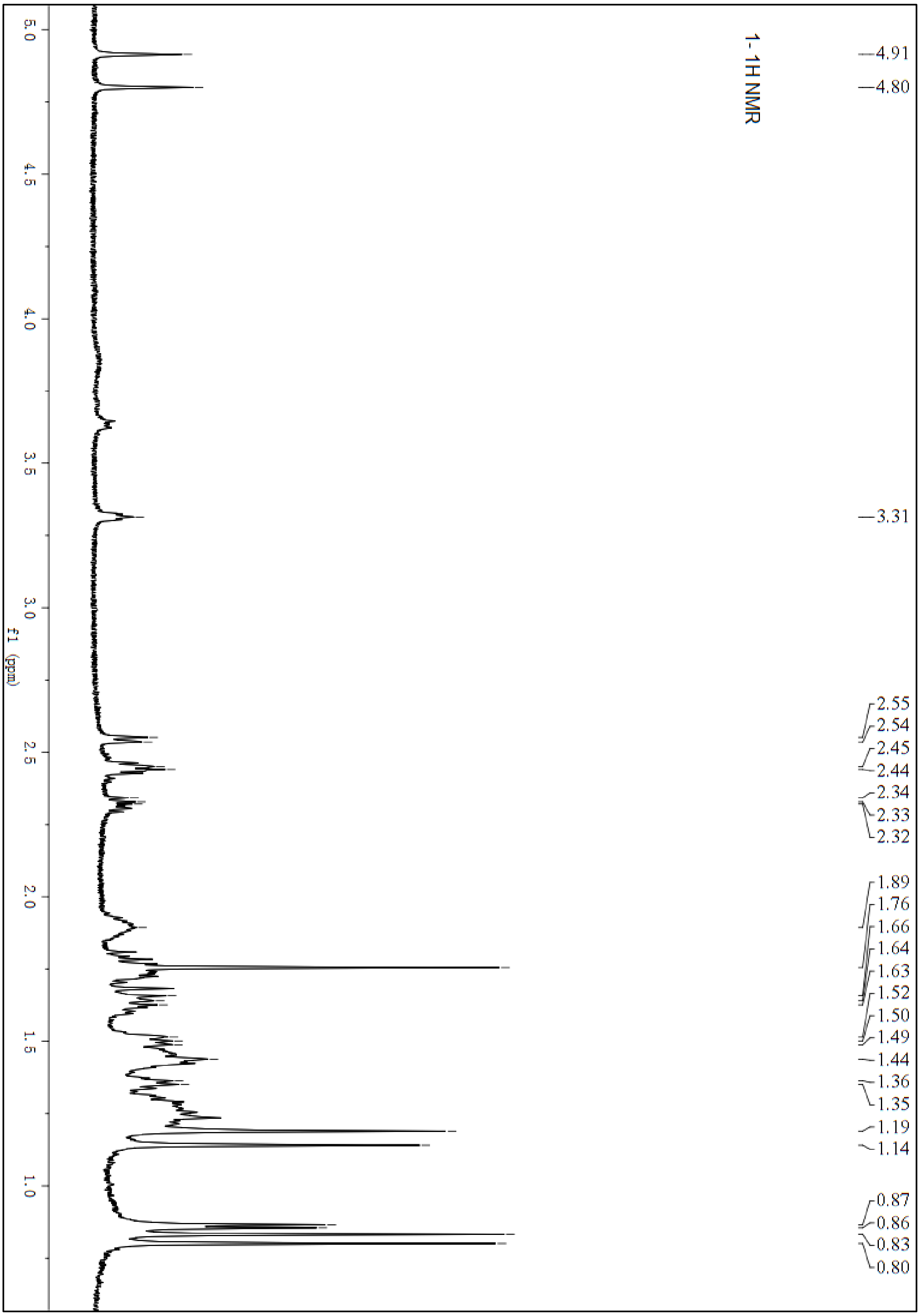

Figure S4. ^{13}C NMR spectrum of Illiciumolide A (**1**) in $CDCl_3$.

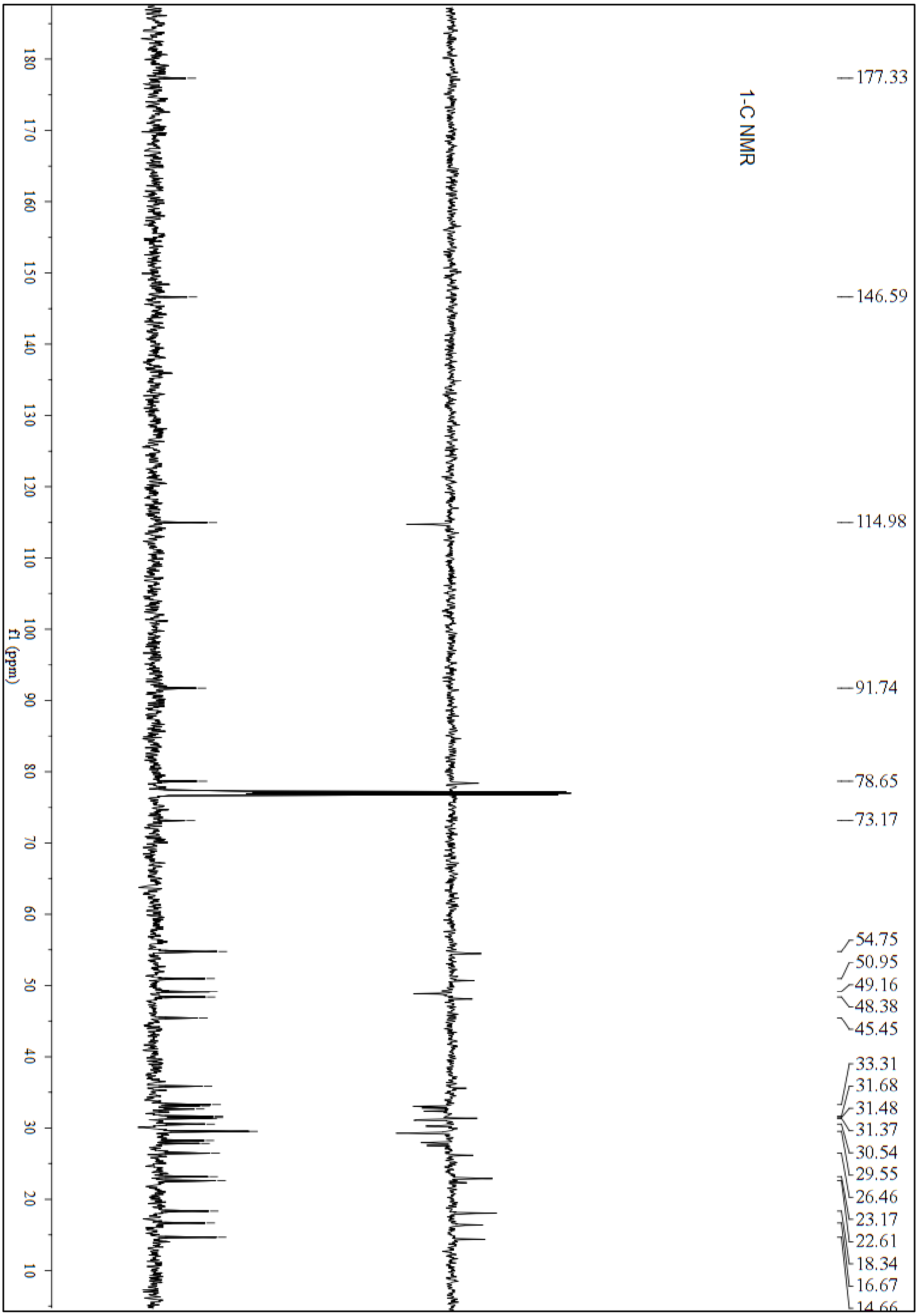

Figure S5. ^1H-^1H COSY spectrum of Illiciumolide A (**1**) $CDCl_3$.

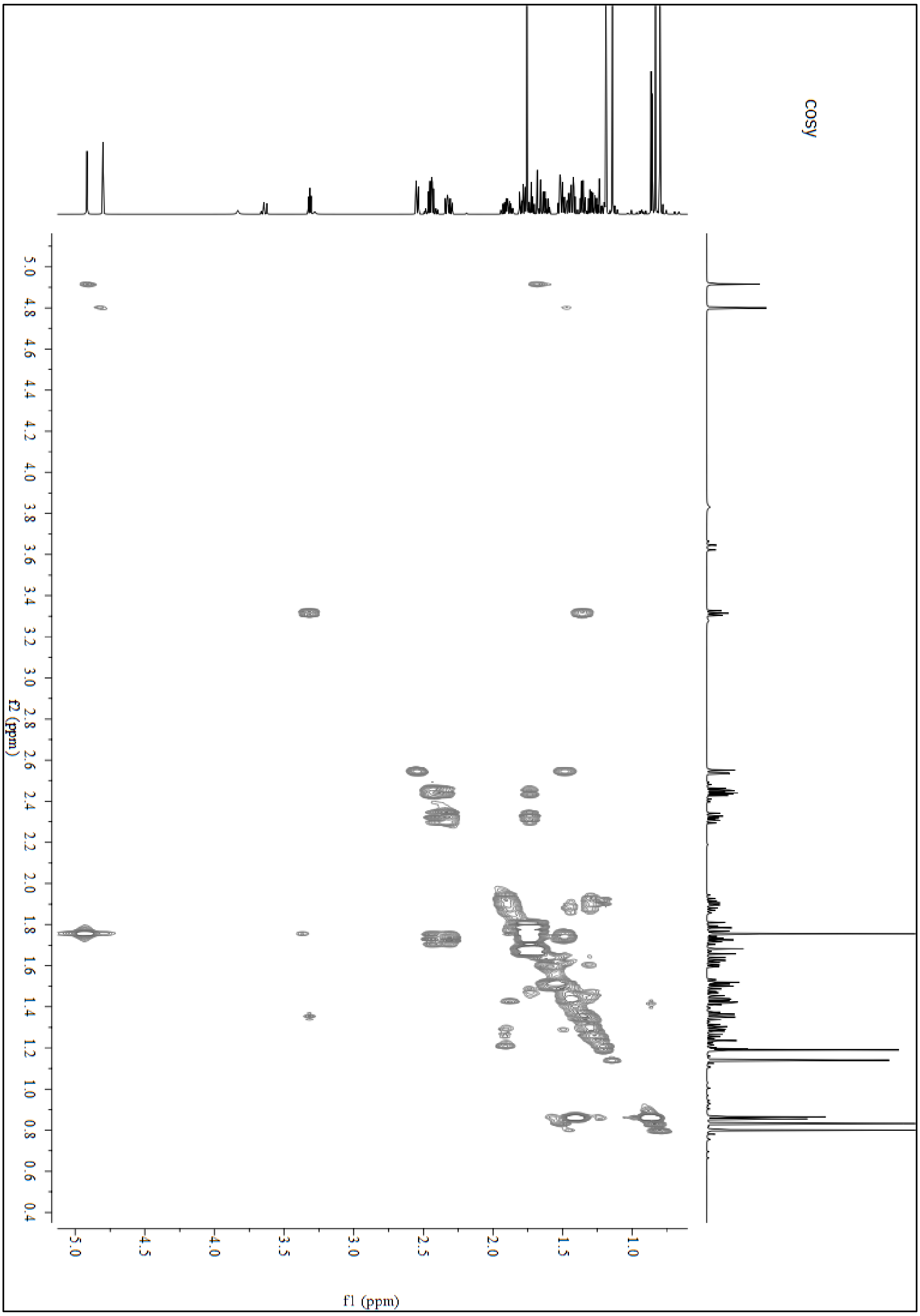

Figure S6. HSQC spectrum of Illiciumolide A (**1**) in $CDCl_3$.

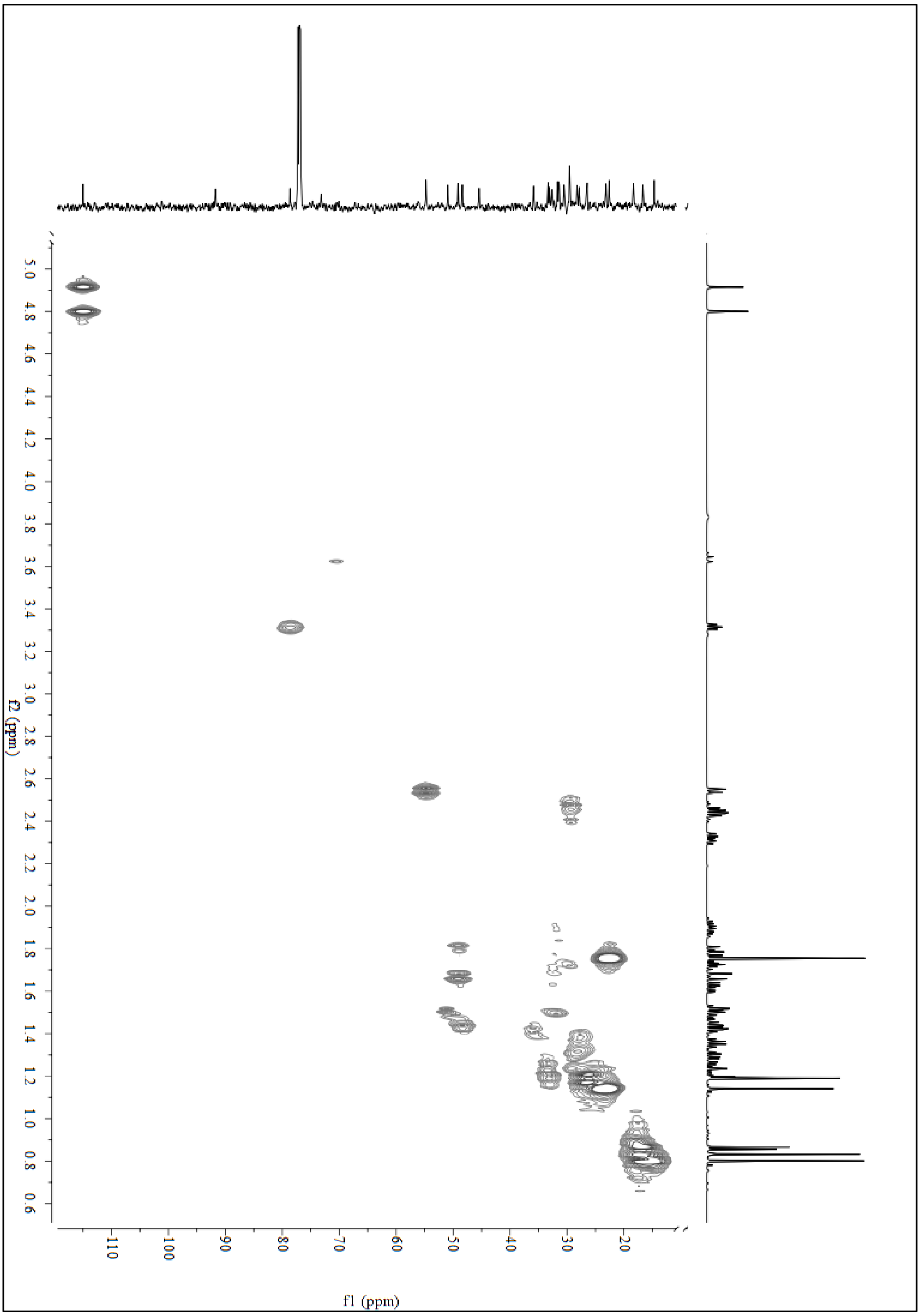

Figure S7. HMBC spectrum of Illiciumolide A (**1**) in $CDCl_3$ (H→C).

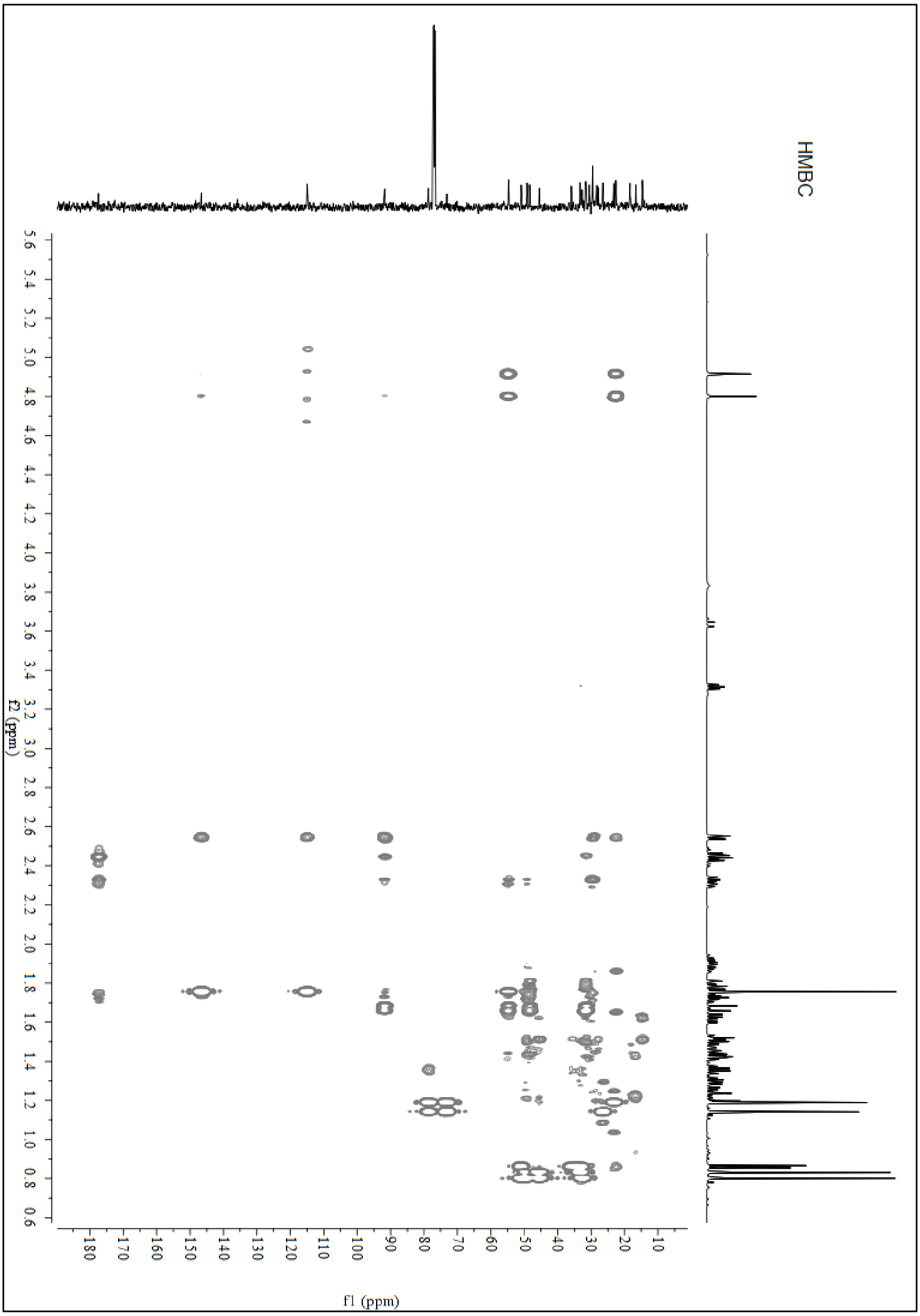

Figure S8. NOESY spectrum of Illiciumolide A (**1**) in $CDCl_3$.

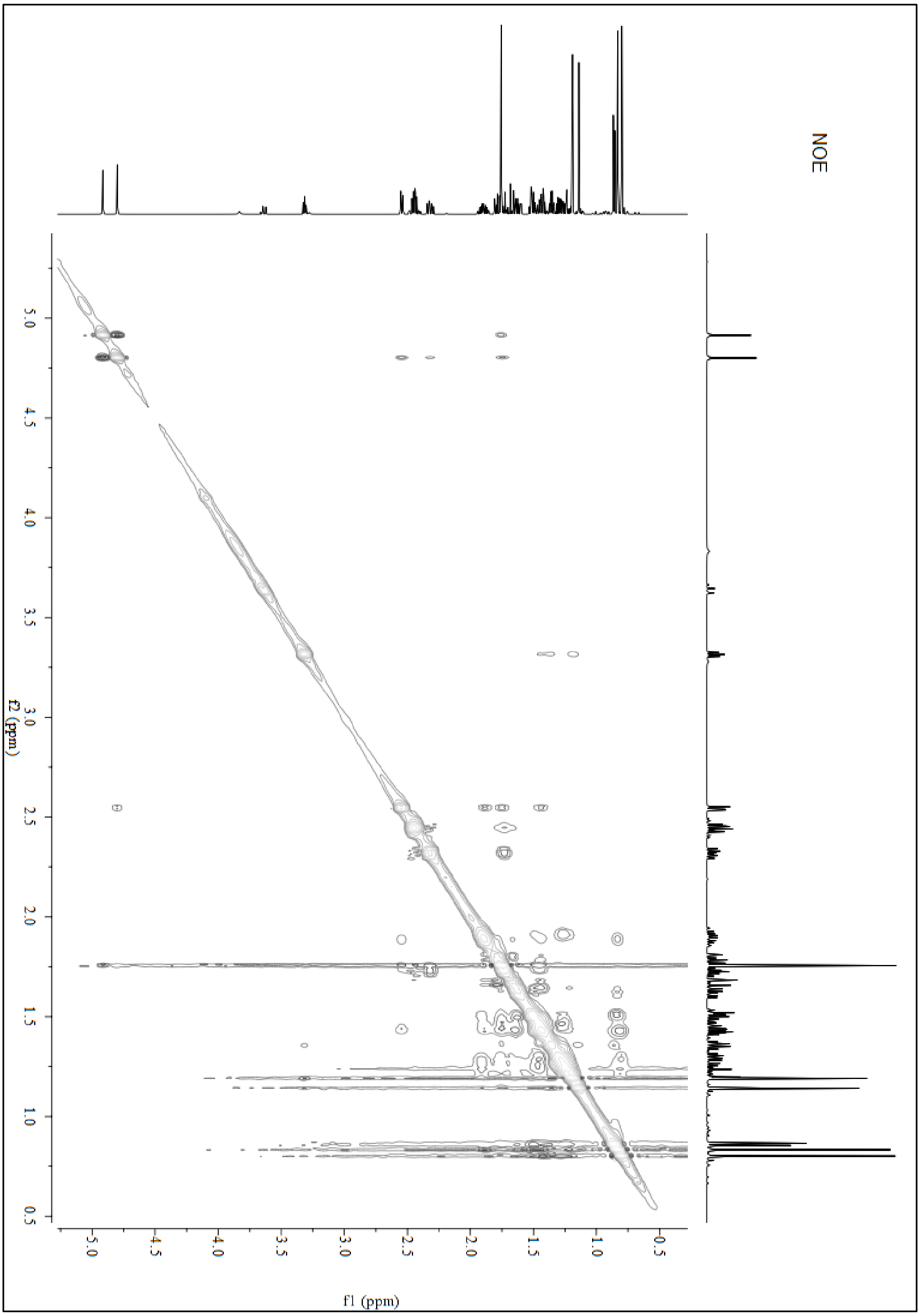

Figure S9. High resolution ESI mass spectrum of Illiciumolide B (**2**).

Qualitative Analysis Report

**Data Filename**	DF-61-POS-1.d	**Sample Name**	DF-61
**Sample Type**	Sample	**Position**	P1-F2
**Instrument Name**	Instrument 1	**User Name**	
**Acq Method**	TEST-POS-03.m	**Acquired Time**	10/16/2012 3:33:45 PM
**IRM Calibration Status**	Success	**DA Method**	ERROR.m
**Comment**			

User Spectra

Fragmentor Voltage	Collision Energy	Ionization Mode
80	0	Esi

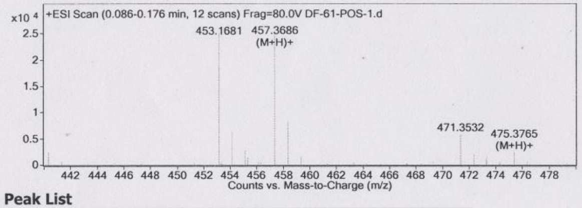

Peak List

m/z	z	Abund	Formula	Ion
121.0509		9962		
274.2752	1	11082		
288.2904	1	19801		
338.2348	1	16874		
338.3424	1	17768		
415.2126	1	21469		
437.1943	1	24199		
453.1681	1	25071		
457.3686	1	25403	C30 H49 O3	(M+H)+
922.0097	1	12404		

Formula Calculator Element Limits

Element	Min	Max
C	0	100
H	0	367
O	0	25
N	0	0

Formula Calculator Results

Formula	Best	Mass	Tgt Mass	Diff (ppm)	Ion Species	Score
C30 H50 O4	TRUE	474.3691	474.3709	3.87	C30 H51 O4	80.6
C30 H48 O3	TRUE	456.3613	456.3603	-2	C30 H49 O3	96.06

--- End Of Report ---

Figure S10. IR spectrum of Illiciumolide B (**2**).

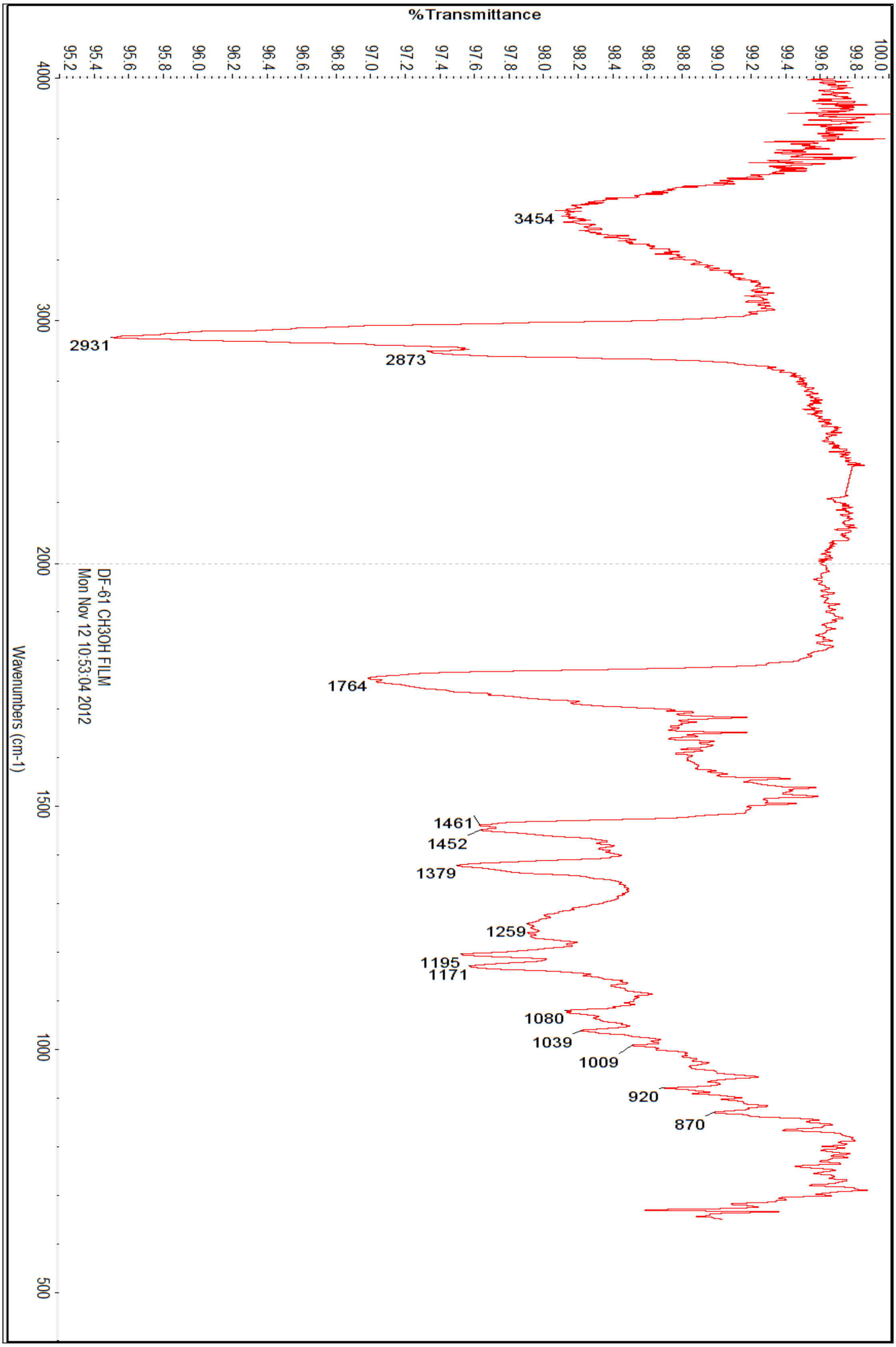

Figure S11 ^{1}H NMR spectrum of Illiciumolide B (**2**) in $CDCl_3$.

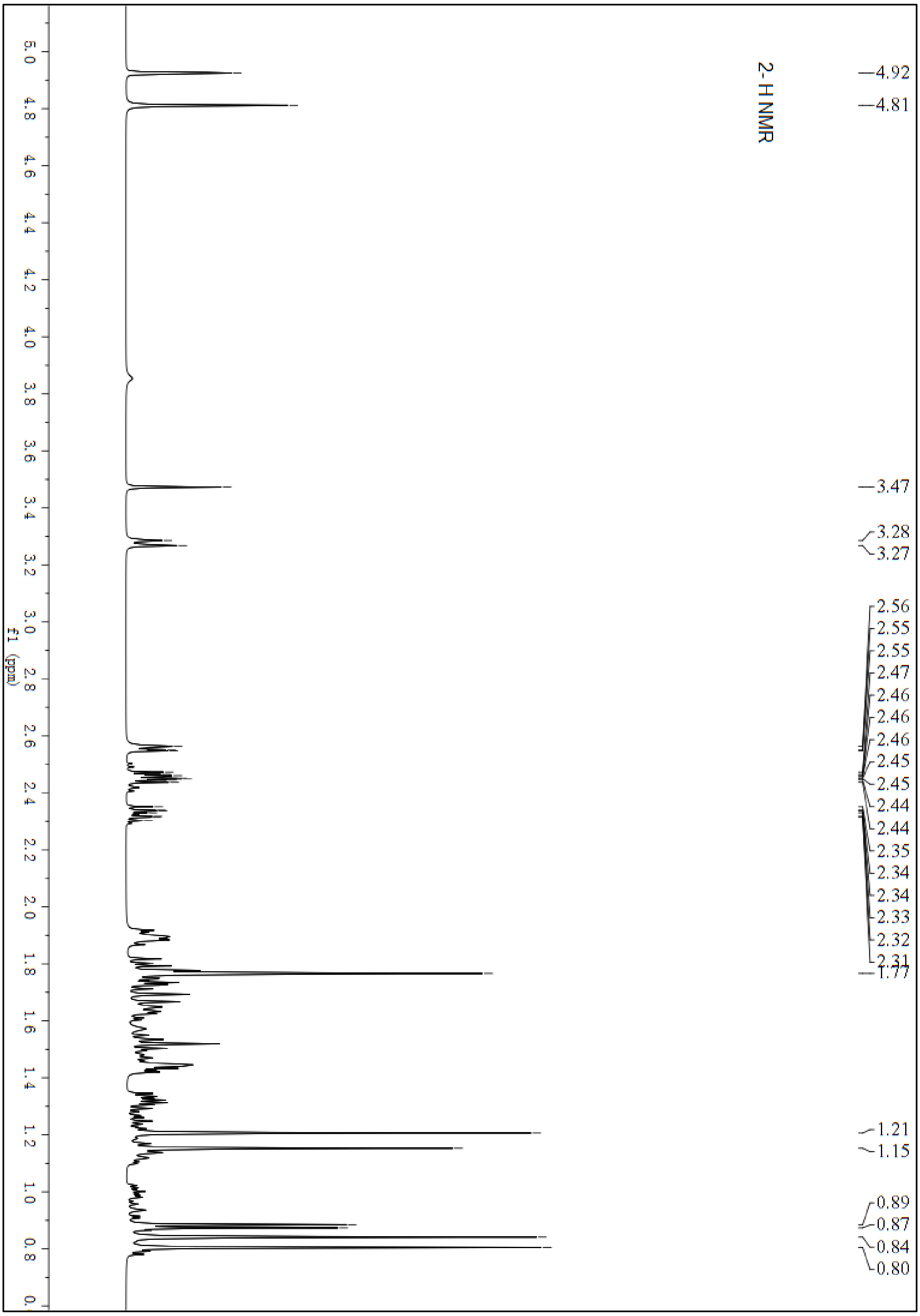

Figure S12. ^{13}C NMR spectrum of Illiciumolide B (**2**) in $CDCl_3$.

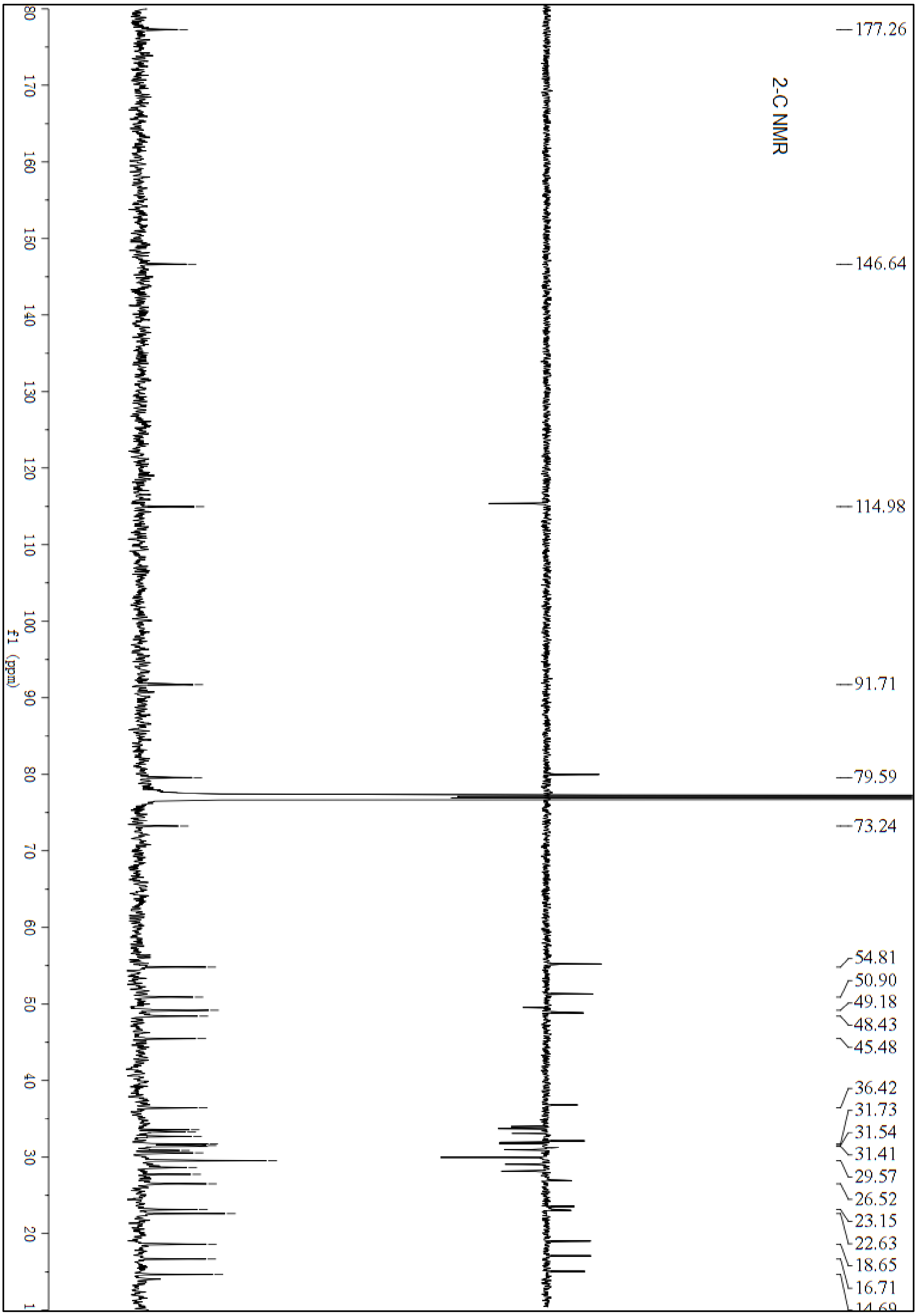

Figure S13. ^{1}H-^{1}H COSY spectrum of Illiciumolide B (**2**) $CDCl_3$.

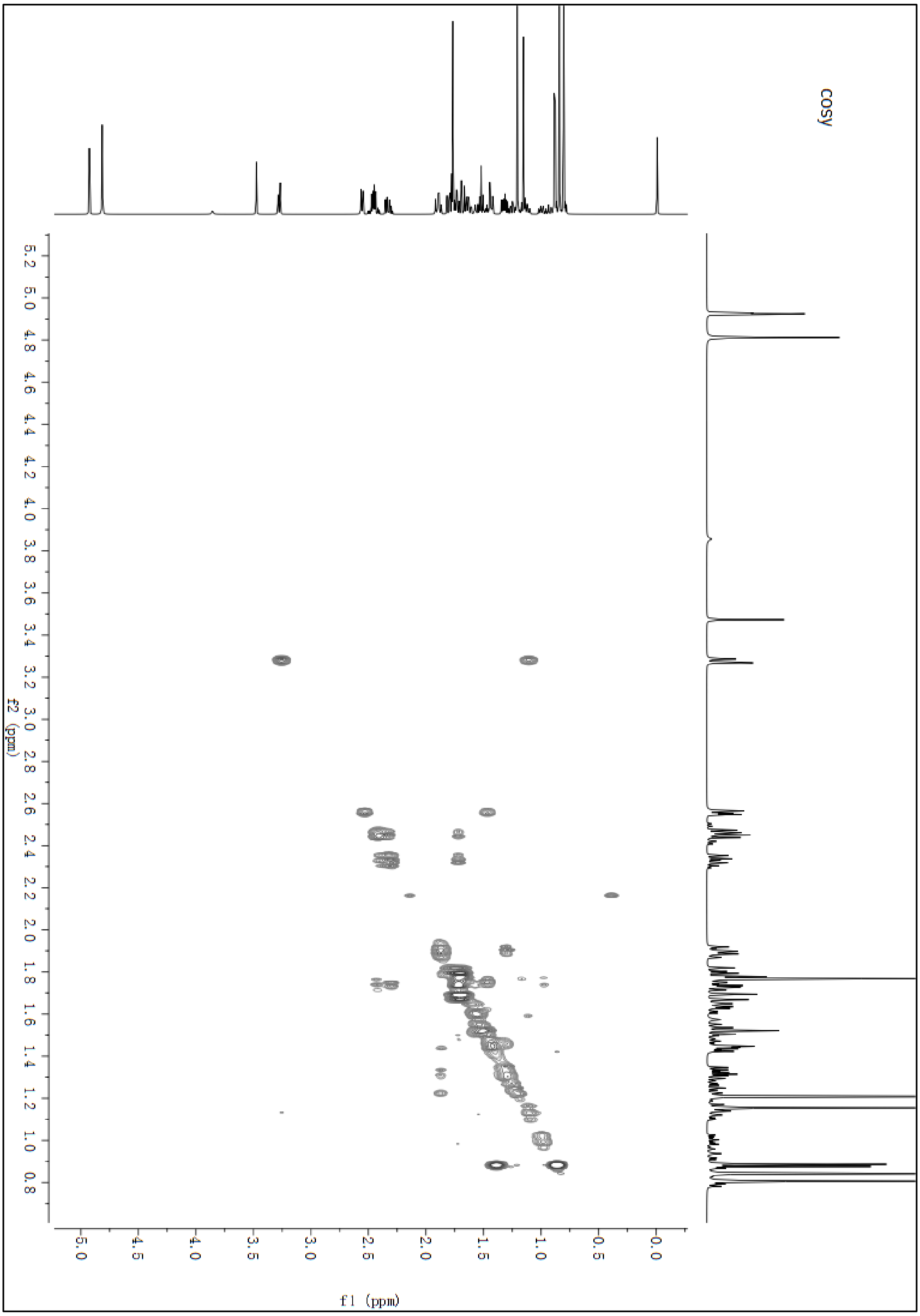

Figure S14. HSQC spectrum of Illiciumolide B (**2**) in $CDCl_3$.

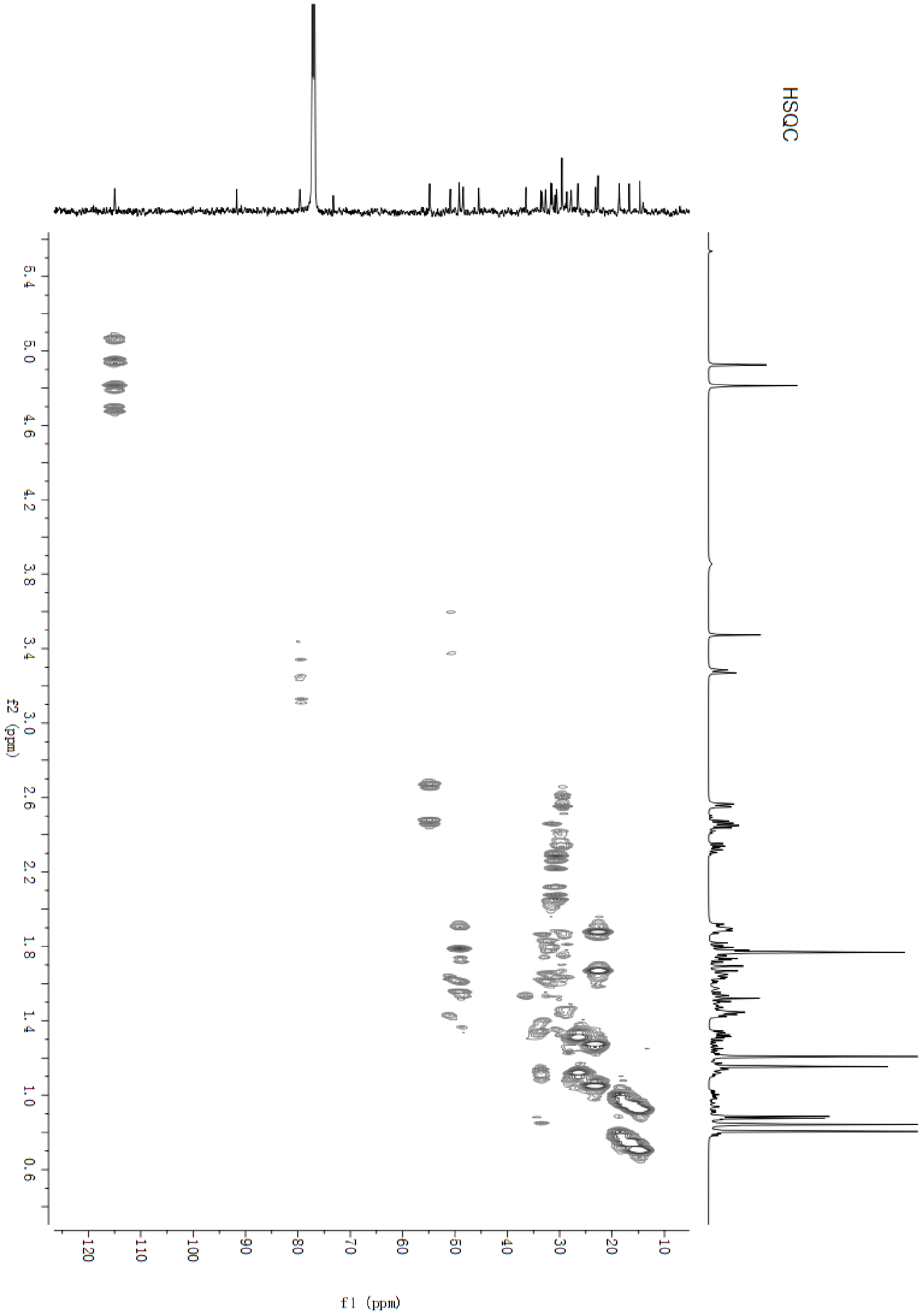

Figure S15. HMBC spectrum of Illiciumolide B (**2**) in $CDCl_3$ (H→C).

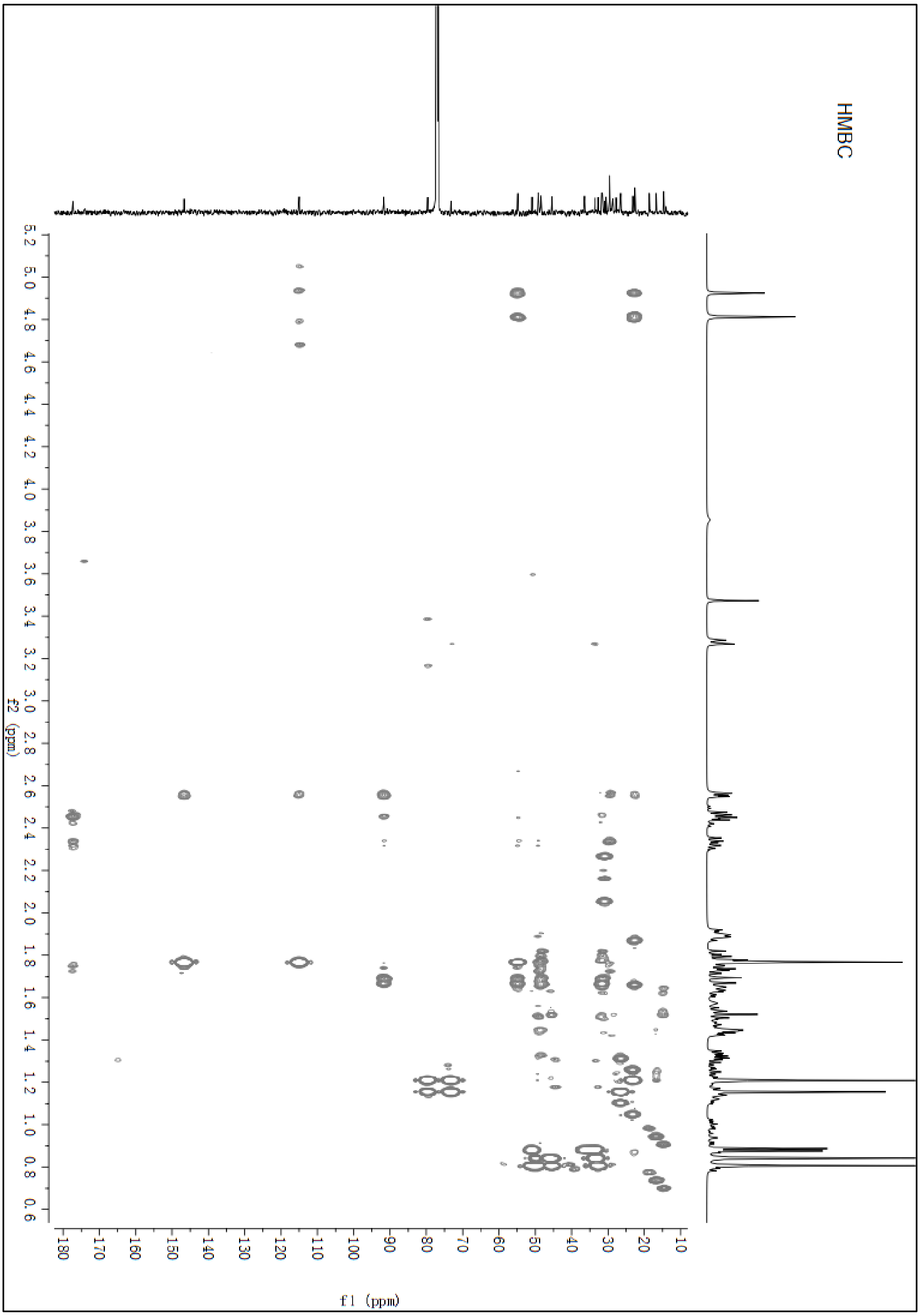

Figure S16. NOESY spectrum of Illiciumolide B (**2**) in $CDCl_3$.

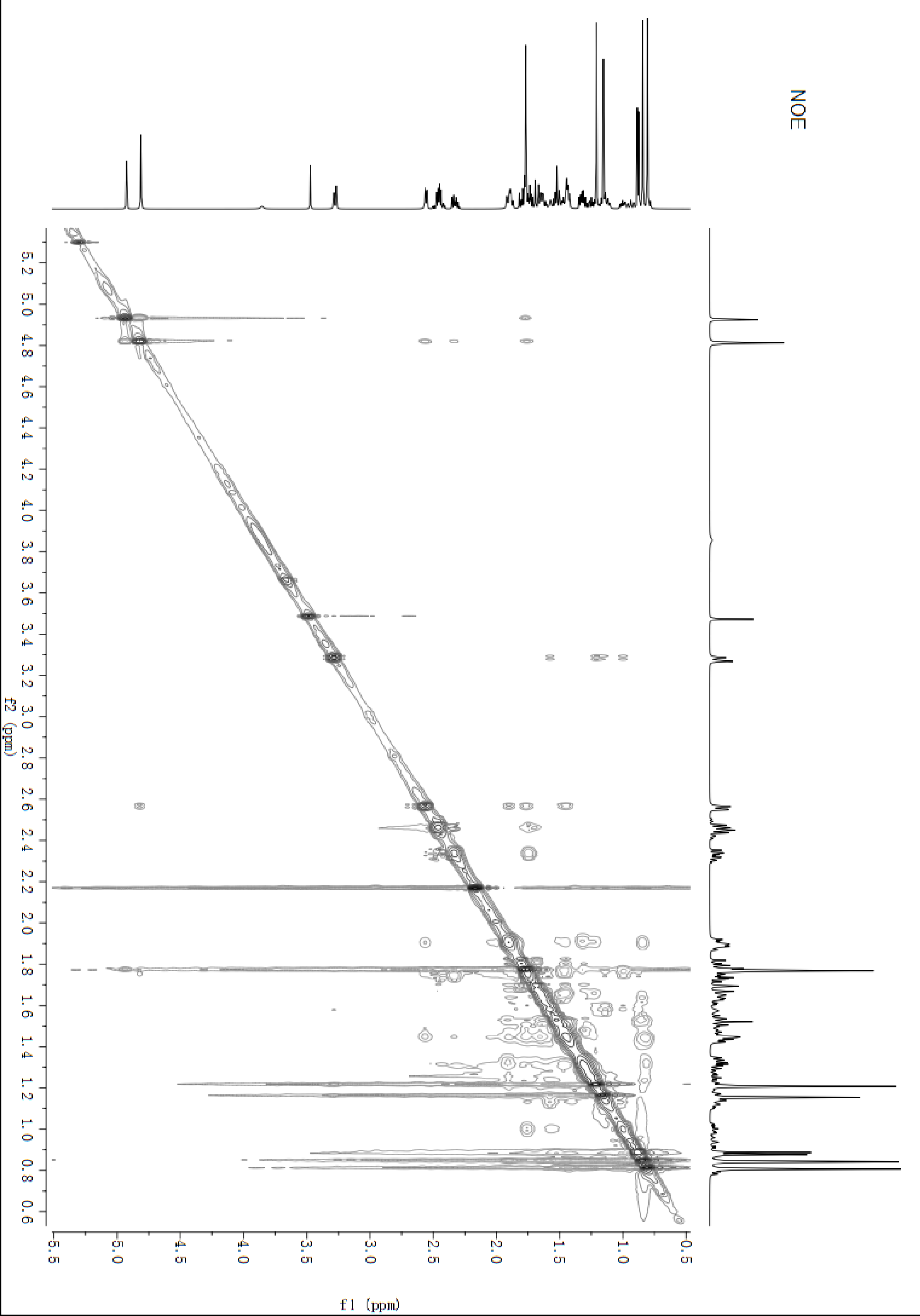

Supplement: Supplementary file 1 — Figure S1: High resolution ESI mass spectrum of Illiciumolide A (1). Figure S2: IR spectrum of Illiciumolide A (1). Figure S3: 1H NMR spectrum of Illiciumolide A (1) in CD3Cl. Figure S4: 13C NMR spectrum of Illiciumolide A (1) in CD3Cl. Figure S5: 1H-1H COSY spectrum of Illiciumolide A (1) CD3Cl. Figure S6: HSQC spectrum of Illiciumolide A (1) in CD3Cl. Figure S7: HMBC spectrum of Illiciumolide A (1) in CD3Cl (H→C). Figure S8: NOESY spectrum of Illiciumolide A (1) in CD3Cl. Figure S9: High resolution ESI mass spectrum of Illiciumolide B (2). Figure S10: IR spectrum of Illiciumolide B (2). Figure S11: 1H NMR spectrum of Illiciumolide B (2) in CD3Cl. Figure S12: 13C NMR spectrum of Illiciumolide B (2) in CD3Cl. Figure S13: 1H-1H COSY spectrum of Illiciumolide B (2) CD3Cl. Figure S14: HSQC spectrum of Illiciumolide B (2) in CD3Cl. Figure S15: HMBC spectrum of Illiciumolide B (2) in CD3Cl (H→C). Figure S16: NOESY spectrum of Illiciumolide B (2) in CD3Cl. [file 942541.f1.pdf]
